# Supplementary material for: Mathematical AI Predicts 40% and 57% Influenza Vaccine Effectiveness Against H1N1 and H3N2, Respectively, for the 2026-–2027 U.S. Season
Source: bioRxiv. 2026 Oct 26:2026.10.22.760980. Preprint. [Version 1] doi: 10.64898/2026.10.22.760980 (PMC13733710; doi:10.64898/2026.10.22.760980)
Supplement: Supplementary file 1 [file NIHPP2026.10.22.760980v1-supplement-1.pdf]

bioRxiv preprint doi: https://doi.org/10.64898/2026.10.22.760980; this version posted October 26, 2026. The copyright holder for this preprint (which was not certified by peer review) is the author/funder, who has granted bioRxiv a license to display the preprint in perpetuity. It is made available under a CC-BY 4.0 International license.

Supplementary Information

A Component-adjusted and within-group associations

The pooled fits combine observations from different subtypes and lineages. Here, we examine whether distance remains associated with VE after allowing separate group intercepts, and whether a relationship is evident within each group separately. Neither analysis established a significant distance–VE association. These results limit the interpretation of the pooled associations, while the small group sizes leave the within-group relationships uncertain.

A.1 Different subtype and lineage intercepts with a shared distance slope

To assess whether distance provided information beyond subtype or lineage, we fitted

$$\mathrm{VE}_i = \alpha_{s(i)} + \beta D_i + \epsilon_i, \tag{16}$$

where $s(i)$ identifies the subtype or lineage of observation i. Each of A(H1N1)pdm09, A(H3N2), B/Victoria and B/Yamagata has a separate intercept $\alpha_{s(i)}$, while β is a single distance slope shared by all four groups. Thus, the model allows different VE levels between groups and estimates the distance–VE relationship after accounting for these mean differences. The topological slope was -13.40 (SE 9.32; 95% CI, -32.8 to 6.0; $p = 0.1651$; model $R^2 = 0.4420$; Table S1). The geometric slope was -17.43 (SE 12.11; 95% CI, -42.6 to 7.8; $p = 0.1649$; model $R^2 = 0.4421$). The two adjusted fits happen to agree closely on both quantities. Neither adjusted slope was significant.

Adjustment reduced the magnitude of the topological slope from -22.84 to -13.40 and the geometric slope from -31.53 to -17.43. This attenuation is consistent with part of the pooled association reflecting differences between viral groups. Both adjusted confidence intervals included zero, so these data did not establish that distance contributed information about VE beyond subtype or lineage identity. However, the wide intervals also allow substantial negative slopes; they do not demonstrate that the within-group relationship is absent.

The adjusted models had higher R^2 than the pooled models, but the extra group intercepts also allow a closer fit to the same observations. This increase describes overall fit and does not establish an additional contribution from distance or improved prediction on later seasons. The common-slope assumption also means that this analysis does not test whether the distance–VE relationship differs between groups; separate exploratory fits are described in Section A.2.

Excluding the figure-derived 2017–2018 B/Yamagata VE changed the adjusted topological slope from -13.40 ($t = -1.44$) to -13.60 ($t = -1.43$). The conclusion was therefore unchanged when this observation was omitted.

A.2 Separate distance–VE fits for each subtype and lineage

We examined whether the pooled distance–VE relationship was also evident across seasons within individual subtypes or lineages. For this exploratory analysis, we fitted a separate intercept and slope for each viral group, rather than imposing the common slope used in Section A.1. The historical

bioRxiv preprint doi: https://doi.org/10.64898/2026.10.22.760980; this version posted October 26, 2026. The copyright holder for this preprint (which was not certified by peer review) is the author/funder, who has granted bioRxiv a license to display the preprint in perpetuity. It is made available under a CC-BY 4.0 International license.

Table S1: Topological pooled and component-adjusted regression estimates on 26 observations. Standard errors are ordinary OLS.

Term	Estimate	SE	95% CI	OLS p	R^2
**Primary: pooled**					
Intercept	76.35	11.35			
Distance D	-22.84	7.05	[-37.4, -8.3]	0.003	0.304
**Sensitivity: subtype fixed effects**					
Distance D	-13.40	9.32	[-32.8, 6.0]	0.165	0.442
Intercept: A(H1N1)pdm09	64.86	16.57			
Intercept: A(H3N2)	54.18	16.73			
Intercept: B/Victoria	69.67	12.98			
Intercept: B/Yamagata	67.19	12.57			

dataset contained seven A(H1N1)pdm09, eleven A(H3N2), five B/Victoria and three B/Yamagata observations.

Within-group analyses did not establish a distance–VE association for any subtype or lineage with either distance. Figure S1a–d shows the topological results. All four topological slope estimates were negative, but their 95% confidence intervals included zero. The small group sizes and wide intervals leave the within-group relationships unresolved. These fits assess whether the pooled association also appears within individual groups and were not used to generate the 2026–2027 forecasts. In particular, the nearly flat H1N1 estimate and the narrow range of B/Yamagata distances provide little evidence of a common pattern within every group. Negative point estimates alone do not establish such a pattern.

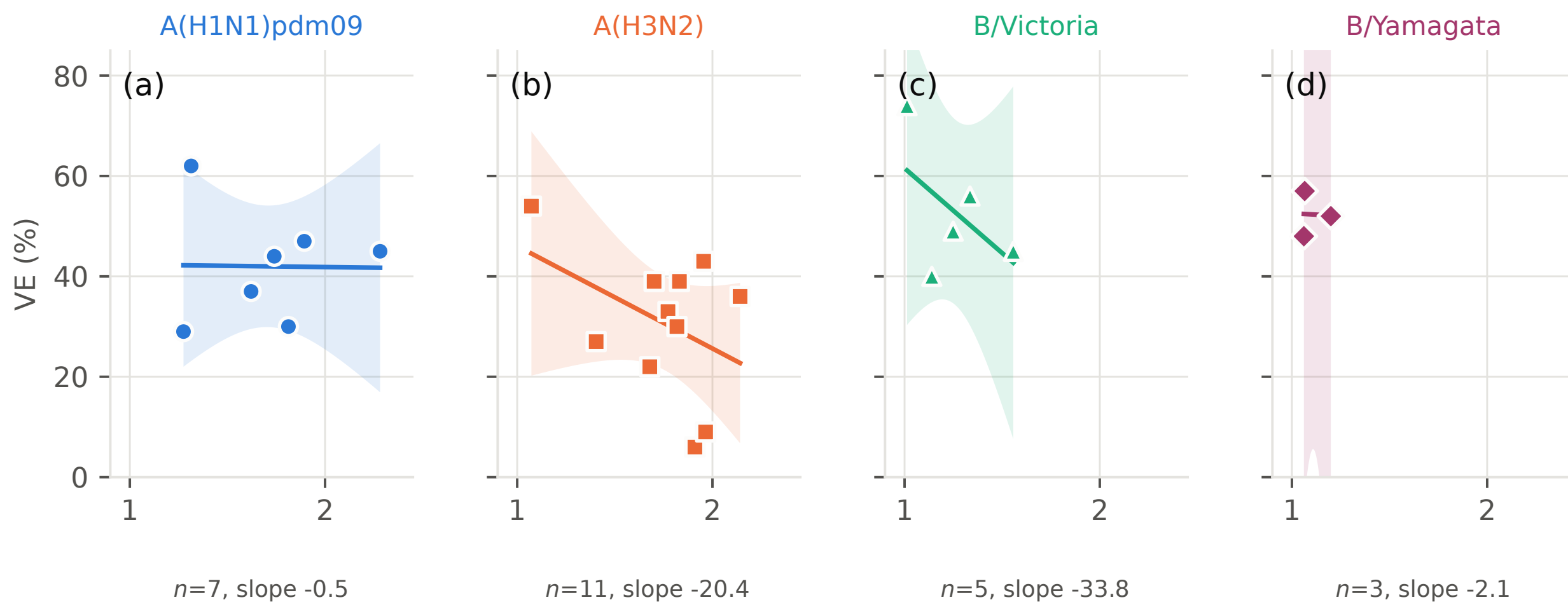

Figure S1: Separate distance–VE fits for each subtype and lineage. (a–d) Historical observations for A(H1N1)pdm09 ($n = 7$), A(H3N2) ($n = 11$), B/Victoria ($n = 5$) and B/Yamagata ($n = 3$), respectively. In every panel, the horizontal axis is the mean August–September topological distance D_A to the vaccine strain, and the vertical axis is clinical VE (%). Each group has a separately estimated intercept and slope. Solid lines and shading show ordinary least-squares fits and 95% confidence bands for mean VE in all four panels. B/Yamagata has only three observations spanning a distance range of 0.14, resulting in a very wide confidence band that extends beyond the displayed VE range. All four slope estimates are negative, but their 95% confidence intervals include zero. These exploratory fits were not used for forecasting.

bioRxiv preprint doi: https://doi.org/10.64898/2026.10.22.760980; this version posted October 26, 2026. The copyright holder for this preprint (which was not certified by peer review) is the author/funder, who has granted bioRxiv a license to display the preprint in perpetuity. It is made available under a CC-BY 4.0 International license.

B Pooled model fits and predictions for later seasons

This section examines how excluding sequence records and VE observations affects the pooled fits, then evaluates predictions for later seasons using only earlier seasons for training. The fitted relationships changed after data exclusions. In historical prediction tests, the geometric pooled model modestly reduced absolute error relative to the historical-mean reference, but prediction intervals remained wide.

B.1 How sequence and VE exclusions changed the pooled fit

Sequence quality checks left 1,285 historical and 87 forecast-season records. The seasonal-human-virus checks excluded 40 historical records: 15 by the clade-prefix rule, 19 by clade 2010.1 and six by the similarity rule when applied in that order. Independently, the similarity rule flagged all 40. Of 1,245 retained historical sequences, 1,226 contributed to the final 26 observations (7 H1N1, 11 H3N2, five B/Victoria and three B/Yamagata). Clinical source checks reduced 28 assembled observations to 26; the two exclusions were fixed before refitting (Section C.3). They changed the pooled topological slope from -19.26 ($R^2 = 0.190$) to -22.84 ($R^2 = 0.304$).

With the same 26 clinical outcomes, using the sequence sets before the seasonal-human-virus exclusions gave slope -9.02 ($R^2 = 0.131$; slope-permutation $p = 0.075$), compared with -22.84 ($R^2 = 0.304$; $p = 0.0033$) after exclusions. Six group means changed; the largest changes were in 2012–2013 H3N2 (11 of 12 records removed) and 2010–2011 H3N2 (two of five removed). These changes show that the pooled fit depends materially on which sequence records and clinical estimates enter the analysis. Its interpretation therefore rests on the documented eligibility rules and source checks. Table S4 provides the final paired data.

B.2 Historical prediction performance of the pooled models

We assessed whether the pooled models could predict VE for a later season using distances and VE observations from earlier seasons. At each test season, distance scales and regression coefficients were re-estimated from the earlier seasons only. Both models were evaluated on the same 19 season–component observations across nine test seasons. Each pooled model was refitted nine times, once per test season. The fit for a season produced predictions for all eligible viral groups in that season. Each prediction corresponds to one clinical VE estimate for a particular season and subtype or lineage. The eligible seasons and inclusion rules are specified in Section C.6.

Table S2 compares prediction errors with a reference that uses the earlier mean VE for the relevant subtype or lineage. This reference asks whether sequence distances add predictive value beyond previous VE levels. The geometric pooled MAE was 11.34 percentage points and the topological pooled MAE was 12.01, against 11.96 for the reference. Both models had lower RMSE than the reference, the topological model the lower of the two.

These summaries rank the approaches differently. The topological model had the slightly higher mean absolute error (12.01 against 11.96) yet was closer to the observed VE in 11 of the 19 tests, against 8 for the reference, a split that an exact sign test does not distinguish from chance ($p = 0.65$); its absolute errors also had the smallest spread of the three (standard deviation 7.97 percentage points, against 9.56 for the geometric model and 9.69 for the reference). The higher mean therefore reflects a few large errors rather than a systematic difference. None of the paired differences in absolute error was distinguishable from zero: topological minus reference $+0.05$ percentage points (paired bootstrap 95% interval -2.97 to $+3.12$), geometric minus reference -0.62 (-2.61 to $+1.52$), and geometric minus topological -0.67 (-3.58 to $+2.53$). With 19 tests, a split of at least 15 to 4

bioRxiv preprint doi: https://doi.org/10.64898/2026.10.22.760980; this version posted October 26, 2026. The copyright holder for this preprint (which was not certified by peer review) is the author/funder, who has granted bioRxiv a license to display the preprint in perpetuity. It is made available under a CC-BY 4.0 International license.

would be needed to reach the conventional significance threshold. These seasons therefore cannot establish an ordering among the two distances and the reference.

The 95% prediction intervals contained the observed VE in 19 of 19 tests for the topological model and 18 of 19 for the geometric model. Their mean widths were 61.0 and 63.0 percentage points, respectively. High coverage therefore accompanied wide uncertainty about individual VE outcomes. Multiple observations came from the same season, and the evidence spans only nine test seasons. These results describe retrospective prediction performance and do not establish reliable prospective forecasts.

Table S2: Prediction accuracy and interval coverage for 19 historical VE observations across nine test seasons. Every row uses the same 19 season–component observations. Each pooled model was refitted for each test season using earlier seasons only. MAE is mean absolute error; RMSE is root mean squared error. Both errors and interval widths are in percentage points (pp). "Observed VE inside PI" counts how many actual VE estimates fall within their predicted 95% intervals. Coverage is this count divided by 19, expressed as a percentage. Thus, the geometric model made 19 predictions, and 18 observed VE estimates fell inside its intervals.

		Prediction error		95% prediction intervals (PI)		
Model or reference	Total predictions	MAE (pp)	RMSE (pp)	Observed VE inside PI (count)	Coverage (%)	Mean width (pp)
Historical-mean reference	19	11.96	15.23	19	100.0	77.9
Topological pooled	19	12.01	14.30	19	100.0	61.0
Geometric pooled	19	11.34	14.67	18	94.7	63.0

Table S3 lists the 19 individual predictions behind these summaries. Absolute errors ranged from 2.5 to 30.1 percentage points for the topological model and from 0.9 to 38.3 percentage points for the geometric model, and both models predicted a higher VE than was observed in 11 of the 19 tests. The geometric prediction was closer to the observed VE in 13 of the 19 tests, but its single largest error was larger than any topological error; the two error measures therefore rank the models differently. Each model was closer to the observed VE than the historical-mean reference in about half of the tests, 11 of 19 for the topological model and 10 of 19 for the geometric model, so the small differences in average error do not correspond to a consistent advantage on individual season–component outcomes.

The largest error for all three approaches occurred in the 2014–2015 A(H3N2) test, which had the lowest observed VE in the data (6%) and was also the earliest test season, fitted on only three earlier observations. This test is the one case in which an observed VE fell outside a prediction interval, the lower limit of the geometric interval being 12.3%. Individual interval widths ranged from 53.9 to 79.2 percentage points for the topological model and from 55.4 to 91.4 for the geometric model. Predictions for single season–component outcomes are therefore compatible with a wide range of VE values, and the historical-mean reference intervals are wider still, extending below zero and above 100% in several early tests.

C Sequence records, VE sources and calculation procedures

This section documents the data and calculations behind the reported results. It first lists the 26 distance–VE observations used to fit the models, followed by vaccine strain records, GISAID sequence sets and checks of the published VE estimates. The remaining subsections describe distance calculations, statistical tests, prediction evaluation and the matching of sampling windows. They

bioRxiv preprint doi: https://doi.org/10.64898/2026.10.22.760980; this version posted October 26, 2026. The copyright holder for this preprint (which was not certified by peer review) is the author/funder, who has granted bioRxiv a license to display the preprint in perpetuity. It is made available under a CC-BY 4.0 International license.

Table S3: Individual predictions for the 19 historical test observations summarised in Table S2. Each row is one clinical VE estimate for one season and subtype or lineage. "Training observations" is the number of earlier season–component observations used to re-estimate the distance scales and regression coefficients for that test season; all rows from the same test season share the same fit. Predictions and intervals are in VE percentage points, and the intervals are 95% future-observation intervals from the fit for that season. They are not constrained to the 0–100% range, so some reference limits fall outside it. † marks the one prediction whose interval does not contain the observed VE. The final rows repeat the summary measures of Table S2, recomputed from the rows shown here.

				Predicted VE (95% prediction interval)		
Test season	Subtype or lineage	Training observations	Observed VE (%)	Topological pooled	Geometric pooled	Historical-mean reference
2014–2015	A(H3N2)	3	6	36.1 (3.3, 68.9)	44.3 (12.3, 76.2)†	44.0 (1.0, 87.0)
2016–2017	A(H3N2)	8	33	38.9 (1.2, 76.6)	39.7 (0.2, 79.1)	36.2 (−18.4, 90.8)
2016–2017	B/Victoria	8	56	47.0 (8.5, 85.5)	53.5 (7.8, 99.2)	49.0 (−21.5, 119.5)
2016–2017	B/Yamagata	8	52	49.5 (9.9, 89.1)	46.5 (6.0, 86.9)	57.0 (−13.5, 127.5)
2017–2018	A(H1N1)pdm09	11	62	48.6 (19.0, 78.2)	46.7 (16.5, 77.0)	45.0 (−1.2, 91.2)
2017–2018	A(H3N2)	11	22	40.9 (11.8, 70.0)	40.7 (10.6, 70.8)	35.7 (0.4, 71.0)
2017–2018	B/Yamagata	11	48	53.9 (22.8, 85.0)	49.3 (18.5, 80.1)	54.5 (14.5, 94.5)
2018–2019	A(H1N1)pdm09	14	44	38.5 (9.7, 67.3)	43.0 (13.7, 72.3)	53.5 (18.2, 88.8)
2018–2019	A(H3N2)	14	9	33.4 (3.7, 63.2)	27.7 (−4.9, 60.4)	33.7 (2.9, 64.5)
2019–2020	A(H1N1)pdm09	16	30	34.7 (4.9, 64.6)	47.7 (19.1, 76.3)	50.3 (15.8, 84.9)
2019–2020	B/Victoria	16	45	41.5 (12.0, 71.0)	26.1 (−3.8, 56.0)	52.5 (15.8, 89.2)
2021–2022	A(H3N2)	18	36	25.7 (−3.4, 54.8)	28.6 (−2.1, 59.3)	30.6 (−0.4, 61.7)
2022–2023	A(H1N1)pdm09	19	47	33.5 (6.3, 60.7)	43.2 (14.8, 71.7)	45.2 (13.7, 76.8)
2023–2024	A(H1N1)pdm09	20	29	49.1 (21.7, 76.4)	47.5 (19.6, 75.4)	45.6 (15.8, 75.4)
2023–2024	A(H3N2)	20	30	36.3 (9.4, 63.2)	30.9 (2.4, 59.3)	31.2 (2.5, 59.9)
2023–2024	B/Victoria	20	74	55.2 (26.7, 83.7)	56.5 (26.6, 86.4)	50.0 (18.6, 81.4)
2024–2025	A(H1N1)pdm09	23	37	40.5 (12.9, 68.2)	40.2 (12.5, 67.9)	42.8 (13.0, 72.6)
2024–2025	A(H3N2)	23	27	46.0 (18.2, 73.9)	34.0 (6.0, 62.0)	31.1 (2.2, 60.0)
2024–2025	B/Victoria	23	40	52.8 (24.2, 81.3)	52.6 (24.1, 81.1)	56.0 (25.1, 86.9)
Mean absolute error (pp)				12.01	11.34	11.96
Root mean squared error (pp)				14.30	14.67	15.23
Observed VE inside interval				19/19	18/19	19/19
Mean interval width (pp)				61.0	63.0	77.9

also identify limitations in record availability, clinical estimates and the coverage of the window comparison.

C.1 Historical distances and VE estimates used to fit the models

Table S4 lists the 26 observations used to fit the historical VE models. Each row represents one season and subtype or lineage and pairs its mean virus–vaccine distance with a published clinical VE estimate. The two distance columns were calculated from the same sequence records; the sequence count identifies how many viruses contributed to each mean. Clinical confidence intervals describe uncertainty in the published VE estimates but were not used as regression weights. The source checks and exclusions are explained in Section C.3; their effect on the pooled fit is reported in Section B.1.

C.2 Vaccine strain records and GISAID sequence sets

Tables S5 and S6 identify the vaccine targets and registered preseason sequence sets. The 23 distinct seed strains supply 43 season–component assignments. Five assignments use records deposited after

bioRxiv preprint doi: https://doi.org/10.64898/2026.10.22.760980; this version posted October 26, 2026. The copyright holder for this preprint (which was not certified by peer review) is the author/funder, who has granted bioRxiv a license to display the preprint in perpetuity. It is made available under a CC-BY 4.0 International license.

Table S4: Historical distance–VE data used to fit the models (26 observations). Mean D_A and D_B are the original-scale Betti-curve and Grassmann distances, rounded to four decimal places, calculated from the same n viruses against the corresponding vaccine strain. VE estimates and references identify the clinical outcomes; 95% CIs are recorded where available and do not enter the regression. Source-specific qualifications are given in Section C.3.

Season	Component	n viruses	Mean D_A	Mean D_B	VE (%)	95% CI	VE source
2010–2011	A(H3N2)	3	1.0728	0.8070	54	[42, 64]	[35]
2011–2012	A(H3N2)	4	1.8310	1.4990	39	[23, 52]	[36]
2012–2013	A(H3N2)	1	1.7012	1.3769	39	[29, 47]	[37]
2014–2015	A(H3N2)	10	1.9102	1.2086	6	[-5, 17]	[38]
2015–2016	A(H1N1)pdm09	8	2.2818	1.1175	45	—	[39]
2015–2016	A(H3N2)	209	1.9550	1.2501	43	—	[39]
2015–2016	B/Victoria	5	1.2487	0.6547	49	—	[39]
2015–2016	B/Yamagata	18	1.0654	0.9243	57	—	[39]
2016–2017	A(H3N2)	99	1.7723	1.1863	33	[23, 41]	[40]
2016–2017	B/Victoria	12	1.3353	0.6340	56	[23, 75]	[40]
2016–2017	B/Yamagata	12	1.1994	0.8882	52	[42, 61]	[40]
2017–2018	A(H1N1)pdm09	33	1.3141	0.8800	62	[50, 71]	[4]
2017–2018	A(H3N2)	166	1.6792	1.1411	22	[12, 31]	[4]
2017–2018	B/Yamagata	38	1.0612	0.7985	48	—	[4]
2018–2019	A(H1N1)pdm09	68	1.7398	1.0124	44	[37, 51]	[41]
2018–2019	A(H3N2)	47	1.9652	1.5111	9	[0, 20]	[41]
2019–2020	A(H1N1)pdm09	47	1.8124	0.9147	30	[21, 39]	[42]
2019–2020	B/Victoria	108	1.5570	1.4553	45	[37, 52]	[42]
2021–2022	A(H3N2)	86	2.1416	1.4638	36	[20, 49]	[43]
2022–2023	A(H1N1)pdm09	39	1.8935	1.0199	47	[1, 72]	[44]
2023–2024	A(H1N1)pdm09	54	1.2748	0.8464	29	[15, 41]	[5]
2023–2024	A(H3N2)	13	1.8180	1.4362	30	[8, 47]	[5]
2023–2024	B/Victoria	4	1.0127	0.5581	74	[65, 81]	[5]
2024–2025	A(H1N1)pdm09	73	1.6211	1.1130	37	[24, 48]	[6]
2024–2025	A(H3N2)	66	1.4044	1.2941	27	[14, 39]	[6]
2024–2025	B/Victoria	3	1.1396	0.7173	40	[12, 59]	[6]

the corresponding season: B/Victoria in 2010–2011, 2011–2012, 2014–2015 and 2015–2016, and H3N2 in 2012–2013. Although strain names match the recommendations, these later records limit strict claims about historical data availability. Target mapping was checked manually.

C.3 Verification of published VE estimates and figure-derived values

Each outcome was checked against its original article before model refitting. Two of 28 assembled observations were excluded: the recorded 2017–2018 B/Victoria value of 76% was not found as a lineage-specific estimate in Rolfes et al. [4], and the recorded 2018–2019 B/Yamagata value of 34% represented combined influenza B rather than that lineage [41].

Twenty-five retained estimates were printed numbers. These include the four 2015–2016 estimates printed in Figure 1 of Jackson et al. [39]. The remaining estimate, 48% for 2017–2018 B/Yamagata, was read from the unlabelled point in Figure 2 of Rolfes et al. [4]. Applying the same reading procedure to four points with printed values recovered 38.1%, 22.0%, 62.0% and 49.9% against 38%, 22%, 62% and 50%, respectively. Omitting the figure-derived estimate left 25 observations and gave the pooled topological fit $\widehat{\mathrm{VE}} = 77.63 - 23.54 D_A$ ($R^2 = 0.301$; slope-permutation $p = 0.0050$),

bioRxiv preprint doi: https://doi.org/10.64898/2026.10.22.760980; this version posted October 26, 2026. The copyright holder for this preprint (which was not certified by peer review) is the author/funder, who has granted bioRxiv a license to display the preprint in perpetuity. It is made available under a CC-BY 4.0 International license.

Table S5: Vaccine target (seed strain) inventory. For each season and component the target is the named WHO/NIBSC/NYMC/CDC reassortant seed strain where one exists on GISAID, egg-passaged, rather than an arbitrary egg-passaged clinical isolate, because the named reassortant is the sequence used to manufacture the vaccine. Accessions and passage histories are as recorded in GISAID. The mapping from each WHO February recommendation to a target sequence was verified manually rather than programmatically.

Vaccine seed strain	GISAID EPI_ISL	Passage	Seasons and components
A/Missouri/11/2025 (IVR-279)	EPI_ISL_20215073	Spf3E1D6	**2026-27 prediction**, A(H1N1)pdm09 (egg)
A/Darwin/1454/2025 (IVR-283)	EPI_ISL_20356066	E2/D7	**2026-27 prediction**, A(H3N2) (egg)
B/Tokyo/EIS13-175/2025	EPI_ISL_20373027	E3	**2026-27 prediction**, B/Victoria (no named reassortant yet)
A/California/7/2009	EPI_ISL_391380	E2E6	2010-11 – 2015-16, A(H1N1)pdm09
B/Brisbane/60/2008 (NIB-58)	EPI_ISL_232491	E4	2010-11, 2011-12, 2013-14 – 2017-18, B/Victoria
A/Perth/16/2009 (NIB-64)	EPI_ISL_70443	E4/E2	2010-11, 2011-12, A(H3N2)
A/Victoria/361/2011 (IVR-165)	EPI_ISL_168948	E6	2012-13, A(H3N2)
B/Wisconsin/01/2010 (BX-41A)	EPI_ISL_83975	EX	2012-13, B/Yamagata
A/Texas/50/2012 (X-223A)	EPI_ISL_132571	E5E8	2013-14, 2014-15, A(H3N2)
B/Massachusetts/2/2012 (BX-51C)	EPI_ISL_402396	E3E7/E1	2013-14, 2014-15, B/Yamagata
B/Phuket/3073/2013	EPI_ISL_291275	E6	2015-16 – 2024-25, B/Yamagata (every season it's the vaccine strain)
A/Switzerland/9715293/2013 (X-247)	EPI_ISL_169603	E4E9+1	2015-16, A(H3N2)
A/Hong Kong/4801/2014 (X-263B)	EPI_ISL_222828	EX/E3	2016-17, 2017-18, A(H3N2)
A/Michigan/45/2015 (X-275B)	EPI_ISL_233044	EX	2017-18, 2018-19, A(H1N1)pdm09
B/Colorado/06/2017	EPI_ISL_276408	E5	2018-19, 2019-20, B/Victoria (and combined B in 2018-19)
A/Singapore/INFIMH-16-0019/2016 (X-307)	EPI_ISL_293533	E5E2E9	2018-19, A(H3N2)
A/Brisbane/02/2018 (IVR-190)	EPI_ISL_20212507	E3/D8/E2	2019-20, A(H1N1)pdm09
A/Victoria/2570/2019 (X-379A)	EPI_ISL_14809235	EX	2021-22, 2022-23, A(H1N1)pdm09
A/Cambodia/e0826360/2020 (X-361A)	EPI_ISL_2378896	EX	2021-22, A(H3N2)
B/Austria/1359417/2021 (BVR-26)	EPI_ISL_5933268	Ex	2022-23, 2023-24, 2024-25, B/Victoria
A/Darwin/9/2021 (NYMC X-369A)	EPI_ISL_5522637	EX	2022-23, 2023-24, A(H3N2) (egg)
A/Victoria/4897/2022 (IVR-238)	EPI_ISL_17086692	E3/E6	2023-24, 2024-25, A(H1N1)pdm09 (egg)
A/Thailand/8/2022 (IVR-237)	EPI_ISL_18372670	E3/D7	2024-25, A(H3N2) (egg)

compared with $R^2 = 0.304$ and $p = 0.0033$ in the primary analysis. The pooled association therefore did not depend on retaining this single figure-derived estimate.

Only point estimates entered the models. Confidence intervals were not available for five observations; among the 21 recorded intervals, median width was 22 percentage points (mean 26.9; range 14–71). The interval held for 2015–2016 H3N2 came from an agency archive rather than the article and is not shown as a primary-source interval in Table S4. The 2022–2023 estimate is late-season rather than a conventional full-season estimate [44]. Clinical estimation uncertainty was not propagated through the regression.

C.4 Word lengths, radius grids and checks of distance calculations

Both distances used HA proteins, the 20 amino acids and word lengths one to three. The protein examples of Hozumi and Wei [20] also use maximum word length three. The Betti-curve implementation used only Betti-0 ($b_k = 0$ in that paper's Equation 16), with radii $\{1, 2, 4, \ldots, N\}$, N being the greater of the two sequence lengths. The Grassmann implementation used 50 log-spaced radii

bioRxiv preprint doi: https://doi.org/10.64898/2026.10.22.760980; this version posted October 26, 2026. The copyright holder for this preprint (which was not certified by peer review) is the author/funder, who has granted bioRxiv a license to display the preprint in perpetuity. It is made available under a CC-BY 4.0 International license.

Table S6: GISAID EPI_SET accessions and DOIs for the preseason searches of each season contributing to the cohort or the forecast. Each DOI resolves to the acknowledgement table for that sequence set. The 2026–2027 entries are the window-closed searches of 9 October 2026 used for the frozen forecast.

Season	Component	GISAID EPI_SET	DOI
2010–2011	A(H1N1)pdm09	EPI_SET_260916yo	10.55876/gis8.260916yo
	A(H3N2)	EPI_SET_260916yt	10.55876/gis8.260916yt
	B (both lineages)	EPI_SET_260916vw	10.55876/gis8.260916vw
2011–2012	A(H1N1)pdm09	EPI_SET_260916er	10.55876/gis8.260916er
	A(H3N2)	EPI_SET_260916mg	10.55876/gis8.260916mg
	B (both lineages)	EPI_SET_260916kv	10.55876/gis8.260916kv
2012–2013	A(H1N1)pdm09	EPI_SET_260916sc	10.55876/gis8.260916sc
	A(H3N2)	EPI_SET_260916fs	10.55876/gis8.260916fs
	B (both lineages)	EPI_SET_260916zm	10.55876/gis8.260916zm
2014–2015	A(H3N2)	EPI_SET_260916eb	10.55876/gis8.260916eb
	B (both lineages)	EPI_SET_260916fh	10.55876/gis8.260916fh
2015–2016	A(H1N1)pdm09	EPI_SET_260916hw	10.55876/gis8.260916hw
	A(H3N2)	EPI_SET_260916cv	10.55876/gis8.260916cv
	B (both lineages)	EPI_SET_260916qy	10.55876/gis8.260916qy
2016–2017	A(H3N2)	EPI_SET_260916ru	10.55876/gis8.260916ru
	B (both lineages)	EPI_SET_260916az	10.55876/gis8.260916az
2017–2018	A(H1N1)pdm09	EPI_SET_260916rg	10.55876/gis8.260916rg
	A(H3N2)	EPI_SET_260916pu	10.55876/gis8.260916pu
	B (both lineages)	EPI_SET_260916ce	10.55876/gis8.260916ce
2018–2019	A(H1N1)pdm09	EPI_SET_260916nr	10.55876/gis8.260916nr
	A(H3N2)	EPI_SET_260916ux	10.55876/gis8.260916ux
	B (both lineages)	EPI_SET_260916nb	10.55876/gis8.260916nb
2019–2020	A(H1N1)pdm09	EPI_SET_260916es	10.55876/gis8.260916es
	B (both lineages)	EPI_SET_260916ps	10.55876/gis8.260916ps
2021–2022	A(H3N2)	EPI_SET_260916wu	10.55876/gis8.260916wu
2022–2023	A(H1N1)pdm09	EPI_SET_261014xz	10.55876/gis8.261014xz
2023–2024	A(H1N1)pdm09	EPI_SET_260916kg	10.55876/gis8.260916kg
	A(H3N2)	EPI_SET_260916aq	10.55876/gis8.260916aq
	B (both lineages)	EPI_SET_260916tr	10.55876/gis8.260916tr
2024–2025	A(H1N1)pdm09	EPI_SET_260916hs	10.55876/gis8.260916hs
	A(H3N2)	EPI_SET_260916du	10.55876/gis8.260916du
	B (both lineages)	EPI_SET_260916xc	10.55876/gis8.260916xc
2026–2027	A(H3N2)	EPI_SET_261009ud	10.55876/gis8.261009ud
	A(H1N1)pdm09	EPI_SET_261009xz	10.55876/gis8.261009xz
	B/Victoria	EPI_SET_261009tf	10.55876/gis8.261009tf

on $[1, 600]$, four channels and rank 10. Both grids depart from the linear schedule $r_i = 4^{k-1}i$ of the source nucleotide implementation, for the reason given in Section 3.2; the upper radius exceeds the longest protein in this dataset (585 amino acids).

The Betti-curve calculation was checked against the published formulas and internal consistency tests; it was not numerically compared with the nucleotide-only reference code. The Grassmann calculation used the reference feature, normalisation, SVD and distance routines of Wang and Wei [23], with the amino-acid word enumeration and radius grid supplied separately. With the reference nucleotide settings, feature blocks matched to machine precision across all four channels and word lengths one to three. Removing rows that are zero in every sequence preserves nonzero features and the subspace distances, provided shared coordinates and the RMS denominator are preserved.

C.5 Permutation tests and intervals for future VE

For the two primary pooled associations, we used two-sided slope tests with 20,000 permutations of the VE labels (NumPy random seed 0). For each permutation, we refitted the regression and compared its absolute slope with the observed absolute slope; the p value was the fraction of permuted slopes at least as large. This gave $p = 0.0033$ for the topological distance and $p = 0.0009$ for the geometric distance. The sequence-exclusion and printed-number-only sensitivity tests used

bioRxiv preprint doi: https://doi.org/10.64898/2026.10.22.760980; this version posted October 26, 2026. The copyright holder for this preprint (which was not certified by peer review) is the author/funder, who has granted bioRxiv a license to display the preprint in perpetuity. It is made available under a CC-BY 4.0 International license.

the same procedure.

We report 95% OLS future-observation prediction intervals. These account for fitted-regression uncertainty and residual variation under the linear model, rather than uncertainty in the mean fitted VE alone.

C.6 Training seasons, test observations and distance scales

The evaluation retained the original common test set for comparison with the historical-mean reference. A season was eligible only if every viral group in that season had an earlier VE observation. The training set also had to contain more observations than the number of previously observed viral groups plus one, ensuring estimable residual variance under the original comparison protocol. These rules were retained when reporting only the pooled models.

The 19 test observations came from 2014–2015 (one), 2016–2017 (three), 2017–2018 (three), 2018–2019 (two), 2019–2020 (two), 2021–2022 (one), 2022–2023 (one), 2023–2024 (three) and 2024–2025 (three). The first three observations, from 2010–2011, 2011–2012 and 2012–2013, provided the initial training set. The 2015–2016 season was excluded as a whole test season because three viral groups first appeared that year; its four observations entered training for subsequent seasons. Thus, seven of the 26 observations contributed to training without being scored as test predictions.

At each test season, we refitted both the regression and distance scales using all eligible observations from earlier seasons. The Betti-curve scales were estimated from their virus–vaccine comparisons. Grassmann scales were estimated from the unique circulating and vaccine protein sequences in the same training data. These scales were then applied to both training and test distances. No sequence from the test season or a later season contributed to scale estimation.

The reference predicted the earlier mean VE for the relevant subtype or lineage. Errors were calculated once per season–component observation, with equal weight across the test observations. MAE averages absolute errors; RMSE takes the square root of the mean squared error. Coverage counts observed VE estimates inside their prediction intervals, and mean width averages the differences between interval limits. The relative MAE reduction is $100(1 - \mathrm{MAE}_{\mathrm{model}}/\mathrm{MAE}_{\mathrm{reference}})$.

For Grassmann scale estimation, the RMS denominator counted all 20^k possible amino-acid words, including zero rows, so later sequences could not alter the row-count convention. A check using the original stored-row denominator gave a geometric MAE reduction of 6.2%, compared with 5.2% under the full-alphabet convention. Both calculations used the same 19 test observations; this check concerns preprocessing. Neither reduction is distinguishable from zero (Section B.2), so the convention does not change the conclusion. The training-only evaluation was implemented retrospectively. Original pooled fits and frozen forecasts retain the project-wide scales described in Section 3.2.

The split follows the seasons in which specimens and clinical outcomes were collected. It does not reconstruct exactly which sequence records or published VE estimates were publicly available at each historical forecast date. Consequently, this is a retrospective evaluation with season-ordered training and testing; prospective performance remains to be established.

C.7 Matching sampling windows and omitting individual pairs or seasons

The topological window comparison used 18 matched observations across eight seasons, with the same vaccine targets and scales in both windows. Scales were estimated from the preseason sequences in the combined 30,464-record collection and applied to both periods. This collection combines the original paired-window search with a later recovery of A(H1N1)pdm09 records, after removing the records flagged by the nonseasonal-virus checks. Correlations were calculated before and after

bioRxiv preprint doi: https://doi.org/10.64898/2026.10.22.760980; this version posted October 26, 2026. The copyright holder for this preprint (which was not certified by peer review) is the author/funder, who has granted bioRxiv a license to display the preprint in perpetuity. It is made available under a CC-BY 4.0 International license.

subtracting group means separately within each window; uncertainty was assessed by resampling whole seasons. This comparison does not use VE labels. Enrollment-period sequences did not enter the primary VE regressions.

The component-demeaned correlation ranged from 0.755 to 0.945 after deleting one pair and from 0.783 to 0.948 after deleting one season. The correlation remained positive and high in each deletion, so no single pair or season was solely responsible for the overall relationship. Within-group correlations were 0.98 for H1N1 ($n = 5$), 0.93 for B/Victoria ($n = 4$), 0.70 for H3N2 ($n = 6$) and -0.30 for B/Yamagata ($n = 3$). In 2018–2019 H3N2, the preseason distance was within 0.06 of its group mean, while the enrollment-period distance was 0.46 above its group mean. Thus, high overall correlation did not imply agreement in every group or season.

The enrollment-period collection contains 29,461 sequences. No GISAID EPI_SET identifier was registered when these searches were performed, so these sequences are not covered by the identifiers in Supplementary Table S6. Their accessions are instead listed by season, viral group and sampling window in `metric_validation/paired_window_accessions.csv` in the repository, from which the corresponding GISAID records and their contributor acknowledgements can be retrieved. These searches nonetheless remain less fully documented than the preseason datasets. The matched seasons were selected by data availability rather than random sampling.
